# Regulation of Fees

**Published:** 1904-09-15

**Authors:** Perry F. Hines

**Affiliations:** Lake Odessa, Mich.


### REGULATION OF FEES.

BY PERRY F. HINES, D.D.S., LAKE ODESSA, MICH.

Read before the Michigan State Dental Association, June 28, 1904.

One of the most important questions confronting our profession to-day is the one which relates to professional remuneration.

At the present time there seems to be a disregard for a uniform and systematized basis upon which to compute professional fees.

Fees are of necessity to be regulated somewhat by the community, the quality of patronage and the fees usually charged by other dentists in that locality, and thus compensation may vary somewhat in different localities; but in every community there will be found at least one dentist who receives better pay, better appreciation for his work, lives better and seems to enjoy life more.

It should be the duty of every dentist, locating in a new practice, to call upon the resident dentists of the town and neighboring towns and ascertain from them, as nearly as possible, the fees received by them for the various dental operations performed in our every-day practice. The resident dentist should also feel it his duty to be frank and inform his prospective competitor of his manner of doing business and the fees which he commonly receives for the various dental operations.

After the incoming practitioner has, by careful inquiry, ascertained as nearly as possible the fees received by his competitors, I would strongly advise him to earnestly strive to give strict attention to his professional duties, and, if possible, do as good or better work than any of them.

Then for the services rendered he should demand a fee equal to or greater than that received by any one of his competitors. By so doing he will receive better appreciation of his work, gain the confidence of his patients, and thus raise himself in the estimation of the people in the community in which he practices.

The receiving of a good fee should be an incentive to him to be more painstaking, to give more time to the work in hand, and thus render to his patients the highest degree of skill within his capability.

He may argue to himself that he cannot demand such fees as Dr. Jones or Dr. Smith, who have been in the place for some time and have built up an established practice, but I wish to say that he *can* and *should*, from the very beginning, demand such fees, providing he renders equally good services. He must not expect, in the beginning, that he will be kept as busy or have as many patients call at his place of business as Dr. Jones or Dr. Smith who have become well established in practice; but there will be some one call upon him to perform some professional duty. This is his chance, his opportunity to demonstrate his ability, and no matter what or how trivial the operation may be, whether it be the extracting of a tooth, the insertion of a gold or an amalgam filling or simply cleaning a set of teeth, he should perform the operation painstakingly and to the very best of his ability. This patient will be his living, speaking advertisement.

We should remember that every person has a friend and some have many friends, and thus when we have performed an operation of real merit and it is appreciated by that patient as such, because of its being so and of his having paid a good price for it, we cannot tell how many more of like character may be attracted to us.

It is the duty of every practicing dentist to spend a part of his time in educating his clients up to a higher standard of dentistry, to give them a clearer insight into the necessity and the manner of preserving the natural dental organs, and the causes which determine the amount of a fee charged by qualified men.

The ability of the dentist is the true standard by which the question of fees should be measured or determined. It is a dentist's duty to charge well for his services. It is a duty because good fees compel good services. Good fees show an appreciation of the dentist's work. They are an incentive to the dentist to do better work, if it is within his power to do so, and to maintain this as a standard of excellence; an incentive to ambition and superior effort, because, in accomplishing something, he is sure of an increased appreciation, evidenced by good fees for good work.

A dentist cannot afford to render good service at a poor price, and he cannot afford to render poor service at any price. By this I mean that whatever the operation he undertakes, he should perform that operation to the best of his ability.

While it is possible for each dentist to maintain a nearly uniform standard of fees, it is not possible to perform for all a uniform grade of work. For, as Hambly says: "In considering this question of compensation, it is necessary to remember that the interests of the patient should be paramount to every other consideration." For instance, if a poor man earning a dollar and a half a day, and having a family to support, should apply to us and we find him suffering great discomfort from an aching tooth, and we know that the tooth could be saved, provided we removed a gangrenous pulp, cleansed, gave it several treatments and filled the roots. Now we might like to advise him to have the tooth so treated, but we realize that this would be expensive to the man, would not at once palliate his pain, and would necessitate his losing time.

In justice to him, and in consideration of his limited means and the imperative need of relief, in order that he may pursue his duties without loss of time, we would extract this tooth and so give relief at once at the least possible expense.

If on the contrary the patient should be a man of means perfectly able to pay any reasonable fee, and whose knowledge of the importance of the teeth is such that he values them most highly and looks with suspicion upon the dentist who would suggest the advisability of removing such a tooth, then we should undertake to save it if there is any possible show for restoring it to health and usefulness.

Here the dentist must use his highest skill, no matter what the expense may be; this patient has the time, the money, and the personal inclination, while the other cannot afford either the time or money. Thus, by saving the rich man's tooth, we do what is best, and by extracting the poor man's tooth, we do what is best for him, from every point of view. Uniformity of price cannot therefore be maintained without taking cognizance of the quality of the clientele.

In estimating the fees on work to be done, sound judgment must be used, and if any great amount of work is to be performed, the dentist will act wisely if he explains to his client somewhat in detail what is necessary to be done and the probable charge or fee that will be demanded for the work; so that they may be prepared for the payment of such fee, and thus avoid unpleasantness when the time comes to pay for the work.

This plan, I think, should be adopted whenever any amount of work is to be or is being performed, unless you are acquainted with your patient and know that the question of the amount of the fee is immaterial to him. An intimation of what the probable charges will be is often a great help in the adjustment of accounts, as it leaves the patient open to make suitable suggestions and gives the operator a better opportunity to learn the desires of the patient and his financial ability, besides being free from any charge of unfairness at the time when the work is finally completed.

After having carefully examined the work to be performed in any case, and having given an estimate of the probable charge, never "jew" or lower such estimate when told by them that they have had a better price or can get the work done more cheaply. Kindly explain to them briefly that the time and skill you propose to give them will be worth all you ask, and that while you would be very glad to work for them, in your practice you could not afford to do the work for less money.

You will find that the people who are always trying to beat you down on your price are shoppers, people who sometimes fabricate and are trying to get something for little or nothing, or are endeavorlng to obtain skilled service service from you at the price of the unskillful. They are quite often fault-finders, and you will, in most instances, be fortunate if they never enter your office the second time.

To make a scale of fees which would be applicable to each and every locality, naming the actual charges which should obtain in any given community, is impracticable; as I have said, the practitioner should acquaint himself with the schedule of fees in use by the other dentists of his locality, and these he should employ or change according to his judgment, being sure to always keep his standard up to that of the highest.

DISCUSSION.

Dr. Essig: In our city we have recently passed through the experience of competition on the fee question. A new dentist came to the city and advertised lower fees than were customary. A considerable number of the other practitioners combined in a public advertisement to meet the cut. The contest continued several months and resulted disastrously to all concerned, and it will be a long time before the profession recovers from this error of judgment if it ever does. The community now thinks that the cut fees were about right and don't want to pay more. In regard to having fees based on ability of patients to pay, I think we are justified in charging patients who are able to pay full fees, because we have so many patients, whom we must serve, that require oftentimes expensive treatment and yet they can't afford to pay even moderate fees.

Dr. Wood: It is a good plan to have one's fees higher than one's competitors, not for the sake of personal aggrandizement, but because it stimulates one to render better service than his competitors. We unconsciously fit our work to the fee we expect to secure for the service. It is better to perform the service and then make the fee on the basis of its value. We are not likely to give the poor man our best service, because we do not expect compensation and we naturally give the rich man the best we can do, because we value his patronage and know he can compensate us. I don't, however, think it is fair to charge a rich man for a poor man's service. I don't allow competitors to influence my fees. I do the best work I can for each patient and get the fee, if possible, that affords me satisfactory compensation. The greatest trouble with this method is that you can not always, in justice to yourself, tell patients the expense of a service before it is rendered.

Dr. Fowler: As a rule I think dentists try to get compensating fees. I try to get all I can, but don't always get all I want, or that I ought to have. I do not think the dentists of Dr. Essig's city did right to try and down the man who came in and cut the fees. It is difficult to correct a crime by committing another. The dentists should have stood with Dr. Essig, and the confidence the community had in them as honorable practitioners would have made itself felt before long, and the fee-cutter would have found his business unprofitable. Several good communities in this State have been almost ruined by this kind of a procedure, so far as any one ever being able to again practice legitimately and honorably in them. If it were possible, I should like to see this Association take some action that would serve to influence its members to do right when such a crisis comes.

Dr. Watson: As a practitioner of a specialty, I find that we have the same difficulties to meet. In fact, I presume there is more shopping in my line than in that of the general practitioner. I can't agree with the speaker who would have the rich patient pay the poor one's bills. Such an action would make us only a means of extorting charity from unwilling benefactors. When I make a concession on fees, I am very sure that my patients so understand it. And I would certainly never lead my rich patients to feel that they have paid some poor man's bill. When I give charity, I want to do it so that I shall benefit my own conscience.

Dr. Douglas: It has always been my policy to strive to do my work as well as I possibly could, and better than any competitor could. I have striven to cultivate this ideal so that it would be a strong temptation, indeed, that would cause me to slight my work because I was not getting as large a fee as I thought it worth. To those able to pay, we should charge a full fee, not an unfair or exaggerated fee, however. When the poor or unfortunate comes for my service, I do the best I can to meet his limitations. It is true some patients who can pay good fees will not, and they are the perplexing ones, and will often try our patience. I make my fee on a standard of my own appreciation of the value of my time, and when it is necessary, discount it always with the patient's knowledge. I have often given the whole of a valuable service to a patient, but that I have counted as a pure benevolence. Where we have an intimate acquaintance with our patients, there is no reason why we should not be able to properly adjust fees equitably.

Dr. Hines: It is impracticable to make a uniform schedule of fees, unless we are serving people of uniform ability to pay, unless we make all fees to fit the least able to pay. A dentist should be benevolent and serve people who can't pay at all. At the same time, for our own satisfaction and business reasons, there must be some general standard which should never be violated without good reason, and then it should be mutually agreed between patient and dentist that a concession has been made for a peculiar condition, but that it, in no case, establishes a precedent. If we don't know our patients, it is only safe or prudent to exact standard fees. It is sometimes a good plan to let strangers know what your services are worth before doing any considerable amount of work for them.

Dr. Snyder: In large cities and almost everywhere in Europe, dentists charge by the hour, while it seems to be the plan in this country to charge by the piece. The colleges are graduating so many new dentists every year that every community in this country has more dentists than can make a living. In Sweden the number of dentists who may practice in a community is limited. Where we would have six or eight dentists here, they would not have more than two. Therefore, for lack of sharp competition, they can more easily maintain a level standard of fees. It would be a good plan to limit the number of dentists in this country.

Dr. Hall: In the old countries where the number of dentists are limited, the amount of services is consequently limited. I recently visited England and I think I never in my life saw so many people with bad teeth. I met a dentist over there who told me of a city, with sixty thousand people living in it, which had only two dentists. Such a city in this country would keep fifty dentists at least busy. The thing to do over there is to let the teeth go until they are irretrievably lost and then extract them and perhaps put in a plate. Decay of the teeth is considered a disease which must run its course, without attempt to interfere. The masses are not educated there as here to care for the teeth, and it seems as though the profession does not care to have them enlightened, since the laws of the country prevent dentists from other countries going in there to practice.

Dr. Wood: I am glad I live in a free country where a man's success depends on his own ability. Any dentist who has taken the care to properly prepare himself and can't find patients enough in this country to make a living ought to get out of the business and take up something more to his liking or in which he can succeed.

Dr. Hoff: It is not equitable in every case to charge the same fee for similar services. It is worth more to work for some patients than it is to do the same work for others. It is worth more to serve a nervous, irritable or petulant patient than it is one who controls himself and does not exhaust the operator. It is not possible to make a set of teeth for every one for the same fee, as some cases are easily fitted and others with great difficulty. This latter fact is probably one of the most important reasons why most dentists do not make artificial teeth. The fee for artificial teeth has come to be established, especially in small cities and towns, on a basis of what the cheapest man in the town is willing to make teeth for. A good operator can't afford to give the time necessary to give the best results at the prices people think are proper, and so this work has fallen to the hands of cheap men and consequently the work is cheap.

I once heard an excellent dentist, who practiced in a large city, tell how he made his fee on a piece of bridge-work he was doing for a very wealthy man. Without any conference with his patient he thought at the beginning of his operation that he ought to get three hundred and fifty dollars for the work. But as he went on with it, he found that it was taking more of his time and skill than he had planned for, and he had, by gradual addition as the days went on, gotten the fee which he intended to charge up to seven hundred dollars. Which was proper enough and justifiable if he was putting the skill and time into the work. But the day before he completed the operation, his wife informed him that their daughter must have a winter coat that would cost fifty dollars. He demurred at first, but finally told her to buy it, while he made up his mind to add fifty dollars to the bridge fee, a dishonorable and unjustifiable action.

